# Experimental colitis in SIV-uninfected rhesus macaques recapitulates important features of pathogenic SIV infection

**DOI:** 10.1038/ncomms9020

**Published:** 2015-08-18

**Authors:** Xing Pei Hao, Carissa M. Lucero, Baris Turkbey, Marcelino L. Bernardo, David R. Morcock, Claire Deleage, Charles M. Trubey, Jeremy Smedley, Nichole R. Klatt, Luis D. Giavedoni, Jan Kristoff, Amy Xu, Gregory Q. Del Prete, Brandon F. Keele, Srinivas S. Rao, W. Gregory Alvord, Peter L. Choyke, Jeffrey D. Lifson, Jason M. Brenchley, Cristian Apetrei, Ivona Pandrea, Jacob D. Estes

**Affiliations:** 1 Pathology and Histotechnology Laboratory, Leidos Biomedical Research, Inc., Frederick National Laboratory for Cancer Research, BG 539, Post Office Box B, Frederick, Maryland 21702, USA; 2 AIDS and Cancer Virus Program, Leidos Biomedical Research, Inc., Frederick National Laboratory for Cancer Research, BG 535, Post Office Box B, Frederick, Maryland 21702, USA; 3 Molecular Imaging Program, National Cancer Institute, Building 10, Room B3B69F, Bethesda, Maryland 20814, USA; 4 Laboratory Animal Science Program, Leidos Biomedical Research, Inc., Frederick National Laboratory for Cancer Research, BG 14D RM 233, 14 Service RD West, Bethesda, Maryland 20814, USA; 5 Washington National Primate Research Center, University of Washington, 1705 NE Pacific Street, Box 357330, Seattle, Washington 98195, USA; 6 Department of Pharmaceutics, WaNPRC, University of Washington, 3018 Western Avenue, Box 357331, Seattle, Washington 98121, USA; 7 Department of Virology and Immunology, Southwest National Primate Research Center, Texas Biomedical Research Institute, 7620 NW Loop 410, San Antonio, Texas 78227, USA; 8 Center for Vaccine Research, University of Pittsburgh, 9044 BST3, 3501 Fifth Avenue, Pittsburgh, Pennsylvania 15261, USA; 9 School of Public Health, University of Pittsburgh, 9044 BST3, 3501 Fifth Avenue, Pittsburgh, Pennsylvania 15261, USA; 10 Department of Microbiology and Molecular Genetics, University of Pittsburgh, 9044 BST3, 3501 Fifth Avenue, Pittsburgh, Pennsylvania 15261, USA; 11 Laboratory Animal Medicine, Vaccine Research Center, NIAID, NIH, BG 40, 40 Convent Drive, Bethesda, Maryland 20814, USA; 12 Statistical Consulting, Data Management Services, Inc., National Cancer Institute at Frederick, Post Office Box B, Frederick, Maryland 21702, USA; 13 Immunopathogenesis Section, Lab of Molecular Microbiology, NIAID, NIH, BG 4 RM 201, 4 Memorial Drive, Bethesda, Maryland 20814, USA; 14 Department of Pathology and School of Medicine, University of Pittsburgh, 9017 Biomedical Science Tower 3, 3501 Fifth Avenue, Pittsburgh, Pennsylvania 15261, USA

## Abstract

Mucosal damage to the gastrointestinal (GI) tract with resulting microbial translocation is hypothesized to significantly contribute to the heightened and persistent chronic inflammation and immune activation characteristic to HIV infection. Here we employ a non-human primate model of chemically induced colitis in SIV-uninfected rhesus macaques that we developed using dextran sulfate sodium (DSS), to directly test this hypothesis. DSS treatment results in GI barrier damage with associated microbial translocation, inflammation and immune activation. The progression and severity of colitis are longitudinally monitored by a magnetic resonance imaging approach. DSS treatment of SIV-infected African green monkeys, a natural host species for SIV that does not manifest GI tract damage or chronic immune activation during infection, results in colitis with elevated levels of plasma SIV RNA, sCD14, LPS, CRP and mucosal CD4+ T-cell loss. Together these results support the hypothesis that GI tract damage leading to local and systemic microbial translocation, and associated immune activation, are important determinants of AIDS pathogenesis.

## Abstract (web summary)

HIV-1 infection in humans and SIV infection in rhesus macaques are associated with mucosal damage to the gastrointestinal tract, microbial translocation and chronic immune activation. Here the authors develop a non-human primate DSS colitis model that recapitulates these aspects of the disease in uninfected rhesus macaques.

## Graphical abstract

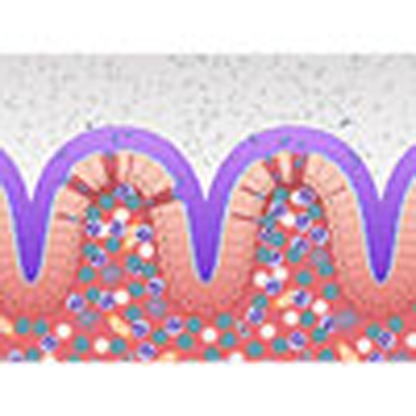

Chronic immune activation is a hallmark of human immunodeficiency virus (HIV)-1 infection in humans and simian immunodeficiency virus (SIV) infection in rhesus macaques (RMs), the most widely used non-human primate (NHP) model of HIV transmission, infection and pathogenesis^1^. Measures of inflammation and immune activation are the best independent predictors of disease progression in HIV-infected individuals, better than either plasma viral loads or peripheral CD4+ T-cell counts^1^. This state of heightened inflammation and immune activation begins early after the acquisition of HIV/SIV and remains high throughout the course of infection, and while typically reduced with initiation of combination antiretroviral treatment still persists in most treated individuals^2^. Persistent gastrointestinal (GI) tract barrier damage and microbial translocation in combination antiretroviral treatment-suppressed individuals is thought to provide a continued source of immune-activating stimuli^3^,^4^,^5^. Importantly, elevated inflammation and immune activation predict non-AIDS-associated morbidities and mortality, even in well-suppressed treated HIV-infected individuals and SIV-infected NHPs^3^,^6^,^7^,^8^,^9^.

The marked persistent activation of the immune system in chronic HIV/SIV infections is a result of numerous factors, but host responses to translocated microbial products from the damaged GI tract are thought to be major contributors^3^,^4^,^10^,^11^,^12^. In support of this hypothesis is the observation that non-progressive natural hosts of SIV infection, which do not manifest progression to AIDS, maintain gut integrity during infection, lack evidence of microbial translocation and do not develop consequent chronic inflammation or immune activation^11^,^13^,^14^. Whereas direct lipopolysaccharide (LPS) administration into chronically SIV-infected African green monkeys (AGMs) increased measures of immune activation and inflammation^15^, conversely blocking microbial translocation in SIV-infected progressive macaques with sevelamer resulted in reduction of inflammation^16^ further establishing microbial translocation as a significant driver for inflammation and immune activation in the setting of lentiviral infections.

The mechanisms underlying damage to the GI epithelial barrier are not completely defined. However, previous work demonstrated that early damage to the GI epithelial barrier occurs during the late acute phase of infection, and once damage has taken place, bacterial constituents that translocate from the lumen of the GI tract perpetuate a state of inflammation resulting in sustained colitis, further exacerbating GI tract damage in SIV-infected RMs^11^.

Loss of barrier function leading to chronic inflammation is not unique to HIV/SIV infections, and has been demonstrated to be an integral part of inflammatory bowel disease (IBD) pathogenesis^17^. Serum from patients with IBD have elevated levels of LPS, LPS-binding protein, circulating C-reactive protein (CRP) and pro-inflammatory cytokines, suggesting that microbial translocation is also linked to systemic immune activation in IBD^18^,^19^,^20^.

While studies from HIV/SIV infections have shown correlative evidence for microbial translocation and GI tract damage and microbial translocation with chronic immune activation, a direct, independent link has not been established. To improve our understanding of the connection between GI tract damage, microbial translocation and systemic inflammation and immune activation as a potential independent driver of characteristic pathologic features seen in HIV/SIV infections, we developed a dextran sulfate sodium (DSS)-induced NHP colitis model in SIV-uninfected RM, clearly establishing links between GI tract damage, local and systemic inflammation and immune activation and characteristic pathologic consequences of HIV/SIV infection in these uninfected animals. The results strongly implicate this process as a key driver of HIV/SIV pathogenesis.

## Results

### Development of an experimental NHP colitis model

To better understand the direct contributions of GI tract damage to microbial translocation and to local and associated systemic inflammation and immune activation, key features of pathogenic HIV/SIV infections, we sought to experimentally induce GI tract damage in NHPs in the absence of lentiviral infection, adopting oral DSS administration, which has been extensively used for rodent experimental colitis models, to NHPs. We chose the oral DSS approach for several key reasons: (i) the ease of administration; (ii) feasibility for translation into a chronic colitis model; (iii) prior uses as a model for IBD in small animals^21^; and (iv) the potential impact on all compartments of the colon, with clinical and histopathological features reflecting those seen in human IBD^21^. In mice, acute colitis has been induced by introduction of 2–5% (w/v) DSS in the drinking water for 4–9 days^21^. This approach has been shown to Induce colitis characterized by bloody stools, weight loss and shortening of the intestine with mucosal ulceration, and neutrophil infiltration^21^,^22^.

Because DSS is believed to be directly toxic to intestinal epithelial cells of the basal crypts and impair the integrity of the mucosal barrier, and because GI tract epithelial damage is a salient feature of pathogenic HIV/SIV infections^5^,^23^,^24^,^25^,^26^, we sought to develop a DSS-induced colitis model in NHPs as the first large animal model of experimentally induced IBD and to evaluate whether GI tract damage in the absence of lentiviral infection is sufficient to lead to microbial translocation, systemic inflammation, immune activation and disruption of the architecture of secondary lymphoid tissues, all pathologic hallmarks of pathogenic HIV/SIV infections. Thus, we set out to establish doses and durations of DSS treatment in NHPs, capable of inducing mild-to-moderate GI tract damage without causing overt clinical symptoms (Supplementary Fig. 1). For dose optimization, healthy SIV-negative RMs (*n*=2) were initially given DSS (1%; w/v) in drinking water for 5 days, which was considered a conservative concentration and duration of DSS based on extensive results in small animal studies using 2–5% (ref. 21). To better control the DSS dose, we administered the DSS for 5 days in non-coloured, fruit-flavoured water supplemented with 1% DSS, changed out three times a day, to achieve a total DSS–water consumption of 100 ml kg^−1^, then supplemented with DSS-free water ad libitum, in accordance with guidelines previously set for NHP daily fluid consumption^27^,^28^. Compared with untreated RMs (*n*=2), RMs given 1% DSS for 5 days had significant clinical signs including bloody diarrhoea with histologic evidence of severe colitis (data not shown). These animals received supportive care with no additional DSS for 8 months to allow colitis and clinical symptoms to resolve before being treated again with the final optimized dosage discussed below. Owing to the initial severity with 1% DSS for 5 days in NHPs, the concentration was reduced fourfold (0.25%; w/v) with the treatment duration extended to 7 or 10 days. A DSS dose of 0.25% for 10 days was determined to be optimal to induce histologically demonstrable mild-to-moderate colitis with few-to-no overt clinical signs.

All animals used in the dose-optimization study were allowed to resolve colitis (rested for 5–8 months) before being treated with the optimized regimen of 0.25% DSS for 10 days. Three days after cessation of DSS, all of the animals (including five healthy untreated control RMs) were euthanized, plasma and peripheral blood mononuclear cells were collected from peripheral blood, along with extensive collection of tissue specimens, including all segments of the colon (that is, caecum, ascending colon, transverse colon, descending colon and rectum), liver and distal lymphoid tissue (that is, axillary lymph nodes, spleen, etc.) for detailed histological assessment.

### DSS-induced colitis in NHPs

**Figure 1:**
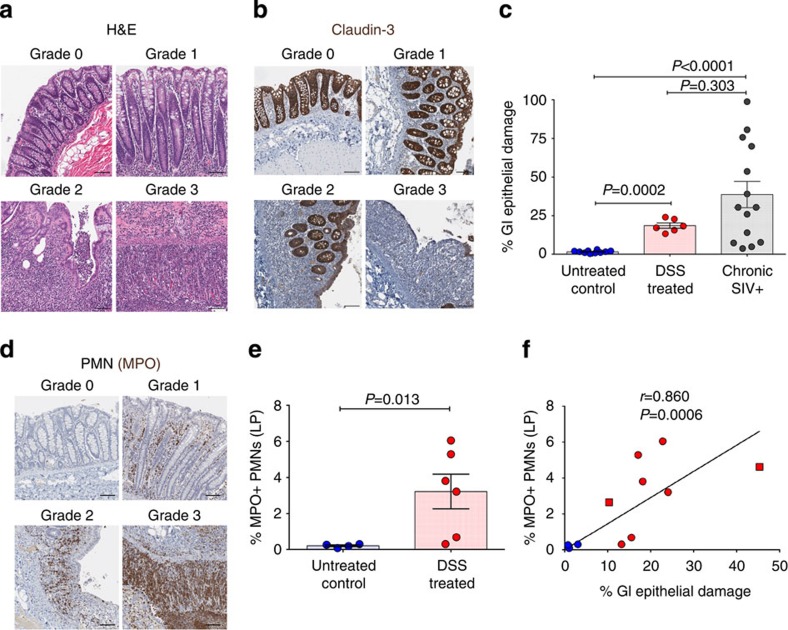
Extent and type of GI tract pathology induced by DSS treatment. (**a**) Representative colon haematoxylin and eosin images showing normal grade 0 (untreated control RM) and grade 1–3 (DSS-treated RM) colitis. (**b**) Representative images of claudin-3-stained colon showing normal grade 0 (untreated control RM) and grade 1–3 (DSS-treated RM) lesions. Notice the strong claudin-3 expression in the colonic epithelium from grade 0 and grade 1, with reduced and absent claudin-3 expression in grade 2 and 3 lesions, respectively. (**c**) Quantification of the percentage of the colon linear length that is damaged (that is, claudin-3 negative). (**d**) Representative images of MPO-stained colon showing the lack of PMN infiltration in normal grade 0 (untreated control RM) colon and the increased PMN infiltrate in grade 1–3 (DSS-treated RM) lesions. (**e**) Quantification of the percent area of the colon (all segments) that is occupied by MPO+ PMNs. (**f**) Direct positive correlation between the extent of GI tract epithelial damage and the magnitude of PMN infiltration into the colon (all segments). Lines are based on linear regression and *r* and *P* values are based on Spearman rank correlation coefficient. *P* values are based on the Mann–Whitney test (**c**,**e**). Untreated control RMs (blue circles) *n*=4 (**e**,**f**) to 10 (**c**), acute DSS-treated RMs (red circles) *n*=6, chronic DSS-treated RMs (red squares) *n*=2, and chronic SIV+ RMs (grey circles) *n*=14. Bar graphs show group means ±s.e.m. with individual animal data points shown. Scale bars=100 μm.

Since damage to the GI tract epithelial barrier resulting in microbial translocation is a distinguishing feature of both pathogenic lentiviral infections^11^,^24^ and IBD, we sought to determine whether DSS-induced colitis in uninfected RM recapitulates salient features of these conditions. First, we performed a comprehensive evaluation of the colon and classified the severity of colitis in DSS-treated RMs according to a pathology grading scale (0–3) (ref. 29) based on observed histologic changes (Supplementary Table 1 and Fig. 1a). Using quantitative image analysis to determine the linear extent of the colon represented by each pathological grade (Supplementary Table 1), here we show that untreated control RMs have on average 98% (range 95–100%) of the colon scored as grade 0 normal tissue, with no lesions or polymorphonuclear neutrophil (PMN) infiltration (Supplementary Fig. 2). The remaining areas of the untreated control RM colon (average 2%; range 0–5%) only contain mild grade 1 lesions with limited PMN infiltration assessed by myeloperoxidase (MPO)+ cells, with no other pathological grades detected in healthy untreated control RMs. In contrast, we observe that only 56% (range 3–87%) of DSS-treated RMs have normal (grade 0) colon, with an average of 36% (range 12–86%) of the colon showing grade 1 lesions (Supplementary Fig. 2). On average, 3% (range 0.1–9%) of the colon in DSS-treated RMs have grade 2 lesions and 2% (range 0–7%) of the colon have grade 3 lesions (Supplementary Fig. 2). Quantification of the density of PMNs (MPO+ cells) within the colon reveal more abundant PMN infiltration in grade 2 compared with grade 1 and in grade 3 compared with grade 2 regions in DSS-treated RMs (Supplementary Fig. 3a), providing quantitative support for our pathological grading classification. Importantly, there is no difference in PMN abundance in normal grade 0 regions of the large bowel in DSS-treated and -untreated control RMs (Supplementary Fig. 3a).

The extent of the pathological damage induced by DSS is multifocal and heterogeneous in extent, with regions of pathology juxtaposed to normal colon as well as regions that contain adjacent foci demonstrating multiple distinct grades of pathology. In comparative assessments by segment of the large bowel, including the ascending, transverse and descending colon and rectum, we show the level of PMNs in each colon segment in DSS-treated animals to be significantly higher than the corresponding segment in untreated animals, with the most extensive GI tract damage (that is, PMN infiltration) seen in the transverse and descending colon (Supplementary Fig. 3b). In addition, we demonstrate that the severity and extent of colitis in DSS-treated, SIV-negative RMs is within the range of colitis seen in chronically SIV-infected RMs. The colon from SIV+ RMs has ∼90% grade 1, 7% grade 2 with no measurable grade 3 lesions and only 3% normal (grade 0). Collectively, in DSS-treated SIV-negative RMs the fraction of the large bowel that is abnormal (grade 1–3) is on average 44% (range 12.5–97.5%), compared with only 2% (range 0–5%) in the untreated control RMs. However, the frequency of abnormal large bowel in chronic SIV+ RMs is on average 96% (range 78–100%).

Next, we evaluate the extent of damage to the epithelial barrier in the colon by immunostaining for the tight junction protein claudin-3 and performing quantitative image analysis to assess the integrity of the GI tract barrier. Following DSS treatment, this analysis shows heterogeneous, multifocal often marked damage to the epithelial barrier, with epithelial disruption and breaches, compared with untreated control RMs (Fig. 1b,c). We compared the extent of colonic epithelial damage in DSS-treated RMs with that seen in chronically SIV+ RMs, and while there is a much greater range of damage in SIV infection, it is not significantly more severe than DSS-treated animals (Fig. 1c).

Because PMN infiltration is associated with damage to the intestinal epithelium in HIV/SIV infections^11^, we evaluated the presence of PMNs in the colon from DSS-treated RMs. GI tract tissues from untreated control RMs have only rare PMNs in the lamina propria, whereas DSS-treated RMs have an abundance of PMNs in regions with pathology grades of between 1 and 3 (Fig. 1d,e). Compared with untreated control RMs DSS-treated animals have significantly increased PMN infiltration collectively in the colon and within each colon segment analysed (Fig. 1e and Supplementary Fig. 3b). Importantly, there is a significant positive correlation between the extent of colonic epithelial damage and the magnitude of PMN infiltration in the colon (Fig. 1f). Collectively, these data demonstrate for the first time a robust model of experimentally induced colitis in healthy SIV-uninfected RMs that recapitulates key features of the GI tract damage and pathology seen in SIV-infected RM.

### GI tract damage results in local immune activation

**Figure 2:**
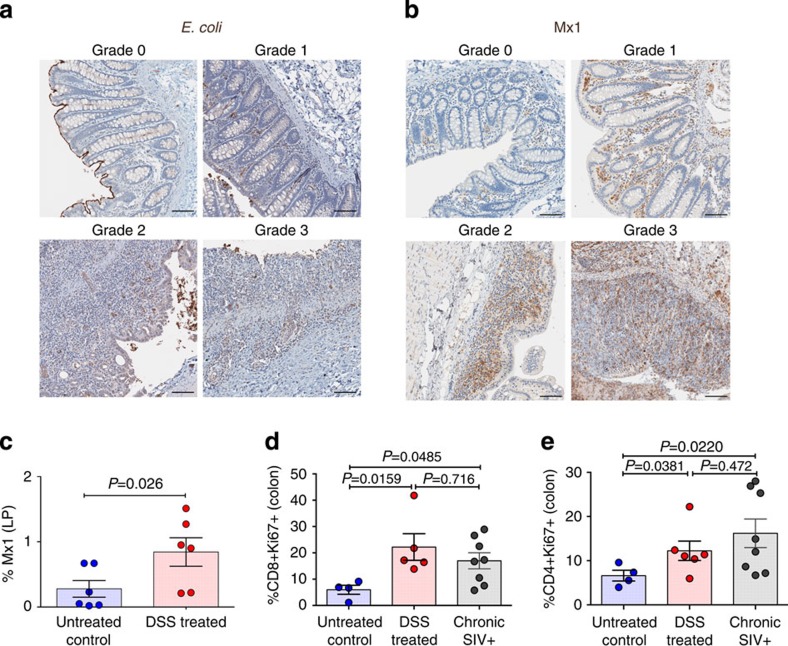
Effect of DSS-induced GI tract damage on local microbial translocation and resulting inflammation and immune activation. (**a**) Representative images of *E. coli*-stained colon showing the lack of microbial translocation in normal grade 0 (untreated control RM) colon and increased presence of bacterial products in grade 1–3 (DSS-treated RM) lesions. (**b**) Representative images of Mx1-stained colon showing limited inflammation in normal grade 0 (untreated control RM) colon and increased inflammation in grade 1–3 (DSS-treated RM) lesions. (**c**) Quantification of the percent area of the colon lamina propria (LP) positive for Mx1. (**d**) The proportion of CD8+ T cells in the colon that express the activation/proliferation marker Ki67. (**e**) The proportion of CD4+ T cells in the colon that express the activation/proliferation marker Ki67. *P* values are based on the Mann–Whitney test (**c**–**e**). Untreated control RMs (blue circles) *n*=4 (**d**,**e**) to 6 (**c**), acute DSS-treated RMs (red circles) *n*=6, and chronic SIV+ RMs (grey circles) *n*=8. Bar graphs show group means ±s.e.m. with individual animal data points shown. Scale bars=100 μm.

To ascertain whether or not GI tract damage resulting from DSS treatment in SIV-negative RMs is associated with local microbial translocation, we looked for the presence of bacterial products within the colon lamina propria^11^. Here we show that colon tissues from untreated animals have only rare microbial products present in the lamina propria (Fig. 2a). In contrast, colon regions from DSS-treated RMs with histopathologic grades 1–3 have abundant evidence of microbial translocation (Fig. 2a).

To determine whether microbial translocation resulting from GI tract damage in DSS-treated RMs leads to local inflammation and immune activation we first evaluated the expression of the interferon-induced GTP-binding protein Mx1 (Mx1) in the colon by quantitative image analysis of the percent area of the colon staining positive for Mx1. Here we show that DSS-treated RMs have significantly elevated Mx1 expression compared with untreated controls, reflecting local inflammation (Fig. 2b,c). As additional markers of inflammation, we evaluated changes in the levels of phosphorylated signal transducer and activator of transcription 1 and 3 (P-STAT1 and P-STAT3), which are transcription factors stimulated by interferons and various pro-inflammatory cytokines. DSS-treated RMs show higher levels of P-STAT1 and P-STAT3 in the colon compared with untreated controls (Supplementary Fig. 4a,b). Similarly, DSS-treated RMs have higher levels of the cellular activation/proliferation marker Ki67 in the colon compared with untreated controls (Supplementary Fig. 5). Next, we specifically assessed the frequency of activated CD3+ T cells by flow cytometry of cells isolated from the colon and here we show that the average percentage of T cell activation (CD4+Ki67+ and CD8+Ki67+) is significantly increased in cells from DSS-treated RMs compared with untreated control animals, with the level of T cell activation in the colon of DSS-treated animals is similar to SIV+ RMs (Fig. 2d,e). Collectively, these data demonstrate that GI tract damage leading to microbial translocation is sufficient to induce robust local inflammatory responses and significantly activate T cell populations within the colon similar to SIV infection.

### GI tract damage results in systemic immune activation

**Figure 3:**
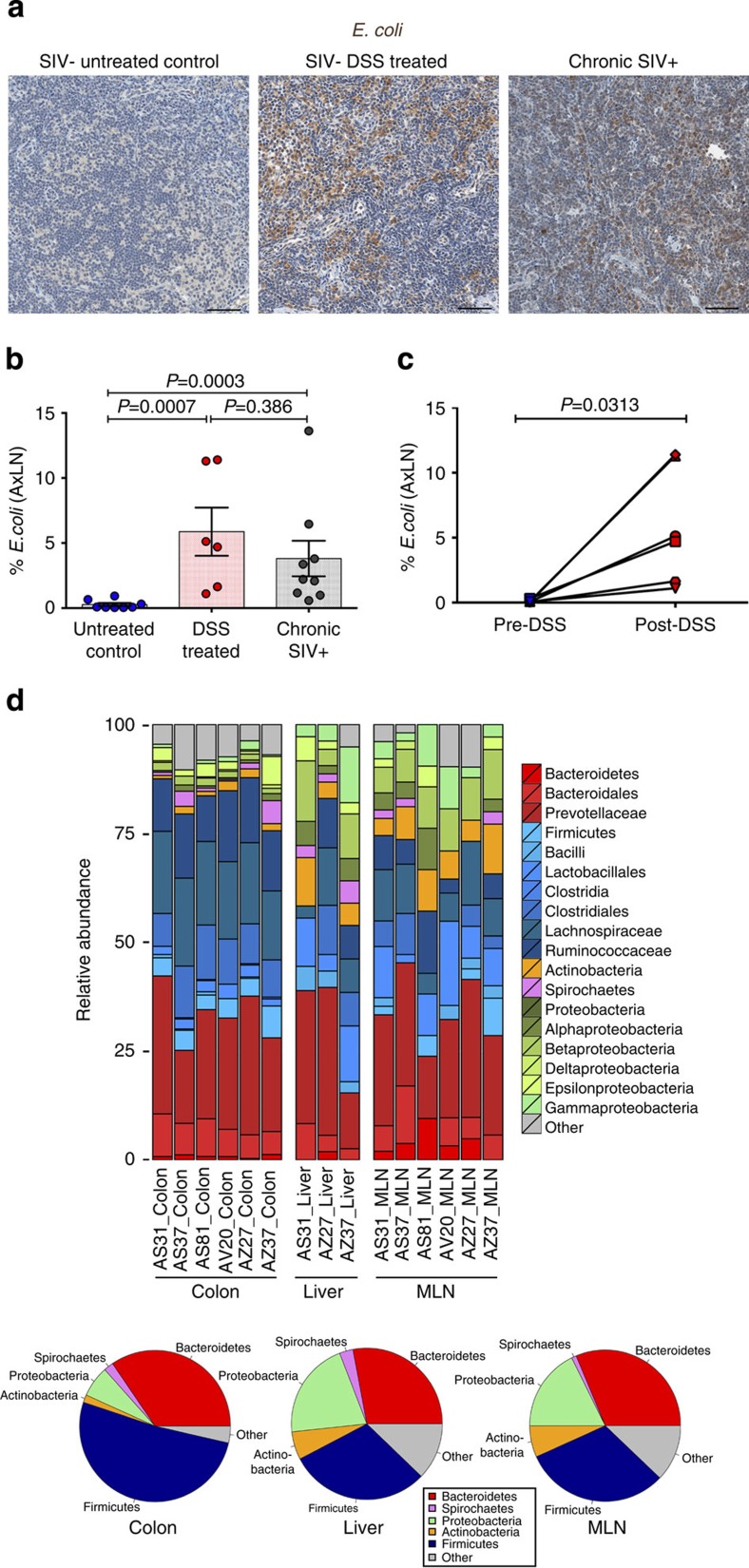
Effect of DSS-induced GI tract damage on systemic microbial translocation within distal tissues. (**a**) Representative images of *E. coli*-stained AxLN showing the lack of microbial translocation in untreated control RM and increased presence of bacterial products in DSS-treated and chronic SIV+ RMs. (**b**) Quantification of the percent area of AxLN positive for *E. coli*. (**c**) Longitudinal analysis of bacterial products (*E. coli*) in peripheral LNs pre- and post-DSS treatment. *P* values are based on the Mann–Whitney test (**b**) or the Wilcoxon matched pairs test (**c**). Untreated control RMs (blue circles) *n*=8, acute DSS-treated RMs (red circles) *n*=6 and chronic SIV+ RMs (grey circles) *n*=9. Scale bars=100 μm. (**d**) Analysis of bacterial communities within the colon and identification of translocating gut flora following DSS treatment analysed for 16S rDNA by 454 pyrosequencing. Bar graphs indicate the relative makeup of the bacterial community in individual animals at each tissue site. Pie charts show the average bacterial community makeup of all animals shown above in colon, liver and MesLN. Analysis of molecular variance with uncorrected pairwise distances calculated from all aligned reads was performed comparing colon and liver (*P*=0.018), colon and MLN (*P*<0.001) and liver and MLN (*P*=0.5). Bar graphs show group means ±s.e.m. with individual animal data points shown.

Chronically HIV+ individuals and SIV+ RMs have measurable amounts of microbial products in the systemic circulation and lymph nodes (LNs) distal to the GI tract^10^,^11^,^30^,^31^. To determine if GI tract damage in DSS-treated SIV-uninfected RMs leads to similar systemic microbial translocation we quantified bacterial products present in axillary LNs (AxLN) using immunohistochemistry (IHC) and quantitative image analysis. Here we show that among SIV-uninfected RMs, untreated control animals have only rare *Escherichia coli* staining within AxLN, whereas DSS-treated RMs have significantly more bacterial products within their AxLNs (Fig. 3a–c). The extent of this microbial translocation in distal LNs in DSS-treated RMs is similar to chronically SIV+ RMs (Fig. 3a,b). These results demonstrate that GI tract damage induced by DSS treatment is sufficient to result in systemic microbial translocation in the absence of lentiviral infection.

To extend these data, we next isolated DNA from snap frozen tissue from DSS-treated RMs and performed 454 pyrosequencing for the bacterial 16S rRNA gene. Using this tool, here we show that bacterial rDNA is detected within the liver and mesenteric LNs (MesLN) of DSS-treated RMs, further confirming that microbial translocation occurs in hosts with a disrupted GI epithelial barrier (Fig. 3d). While rDNA found in distal tissues is consistent with derivation from intestinal flora, the relative proportions of select bacterial phyla within liver and MesLN are distinct from those seen within the colon showing an increased proportion of the phyla Proteobacteria and Actinobacteria (Fig. 3d). This finding suggests that these subsets of microbes may preferentially translocate to distal sites when epithelial integrity is compromised, similar to what we have observed in SIV-infected macaques^32^.

**Figure 4:**
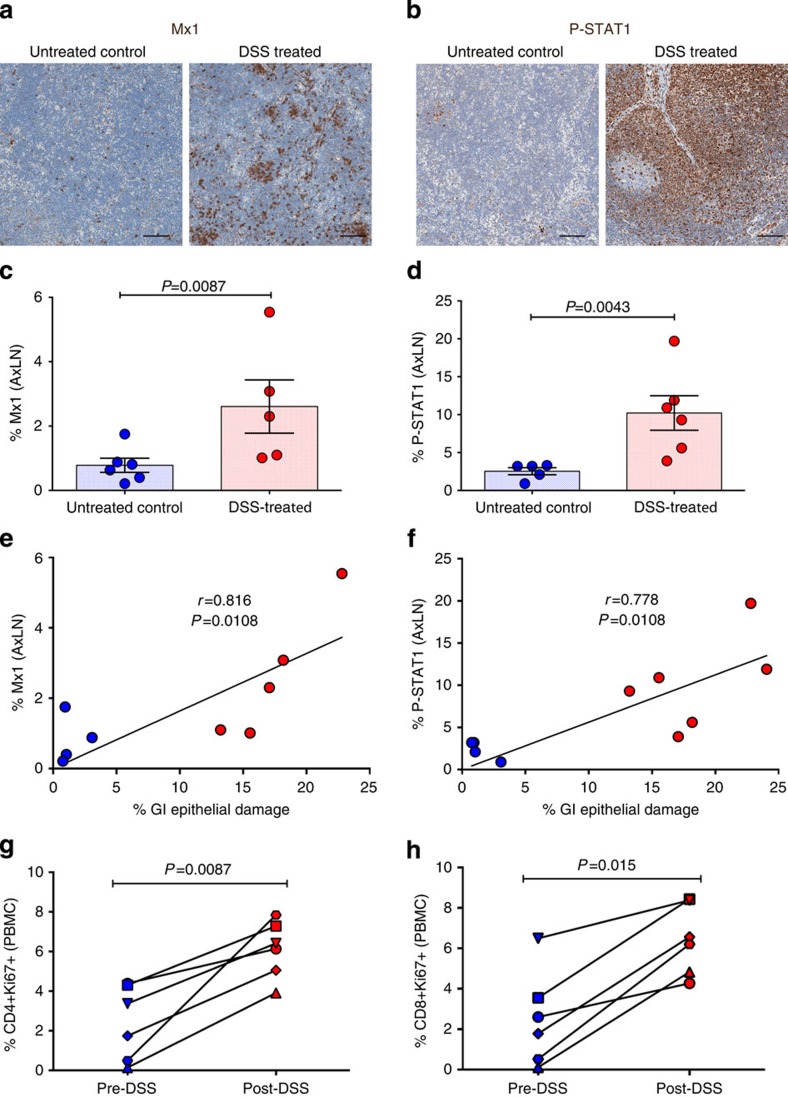
Effect of DSS-induced GI tract damage on systemic inflammation and immune activation within the distal AxLN. Representative images of (**a**) Mx1- and (**b**) P-STAT1-stained AxLNs showing limited inflammation in untreated control RMs and increased inflammation in DSS-treated RMs. Scale bars = 100 μm. Quantification of the percent area of the AxLN positive for (**c**) Mx1 and (**d**) P-STAT1. Direct positive correlation between the extent of GI tract epithelial damage and the magnitude of (**e**) Mx1 and (**f**) P-STAT1 expression in AxLNs. Lines are based on linear regression and *r* and *P* values are based on Spearman rank correlation coefficient. Longitudinal analysis of (**g**) CD4+Ki67+ and (**h**) CD8+Ki67+ in the peripheral blood mononuclear cells (PBMC) pre- and post-DSS treatment. *P* values are based on the Mann–Whitney test (**c**,**d**) or the Wilcoxon matched pairs test (**g**,**h**). Untreated control RMs (blue circles) *n*=4 (**e**–**h**) to 6 (**c**,**d**) and acute DSS-treated RMs (red circles) *n*=6, with individual shapes representing individual animals (**g**,**h**). Bar graphs show group means±s.e.m. with individual animal data points shown.

Next we sought to determine whether the GI tract damage leading to systemic microbial translocation observed in DSS-treated RMs is sufficient to lead to systemic inflammation and immune activation. Consistent with systemic inflammation, here we show that IL-8 is significantly elevated in the plasma after DSS treatment (Supplementary Fig. 6a). In addition, we find within distal AxLN multifocal regions with elevated expression of Mx1, P-STAT1, and Ki67 and PMN cells (Fig. 4a,b and Supplementary Fig. 6b,c), with significantly higher Mx1+ and P-STAT1+ cells in AxLNs of DSS-treated RMs compared with untreated controls (Fig. 4c,d). The magnitude of Mx1 and P-STAT1 expression in AxLN is directly correlated to the extent of GI tract damage (Fig. 4e,f). Finally, we find that activation in peripheral blood T cells (CD4+Ki67+ and CD8+Ki67+) is significantly increased after DSS treatment compared with pretreatment time points (Fig. 4g,h), demonstrating that GI tract damage leading to microbial translocation is sufficient to systemically drive both inflammation and immune activation.

### Longitudinal evaluation of GI tract damage using MRI

Next, we sought to determine whether we could extend our NHP colitis model by (i) inducing chronic colitis with multiple, repeated DSS treatment cycles, and (ii) monitoring progressive GI tract damage and inflammation with a novel, noninvasive, longitudinal magnetic resonance imaging (MRI) approach correlating MRI findings with histological analysis. Since IBD is associated with alterations and dysfunction of the intestinal microvasculature with leakage from the colonic microvasculature at focal inflammatory regions^33^,^34^, we explored the utility of a gadolinium-based MRI contrast agent (gadofosveset trisodium; Ablavar) that reversibly binds to serum albumin and has been used clinically for magnetic resonance angiography to diagnose vascular diseases^35^,^36^,^37^,^38^. We monitored the accumulation and intensity of gadofosveset trisodium in GI tract tissues by MRI in two healthy SIV-negative RMs treated with three to four cycles of DSS (Supplementary Fig. 7).

**Figure 5:**
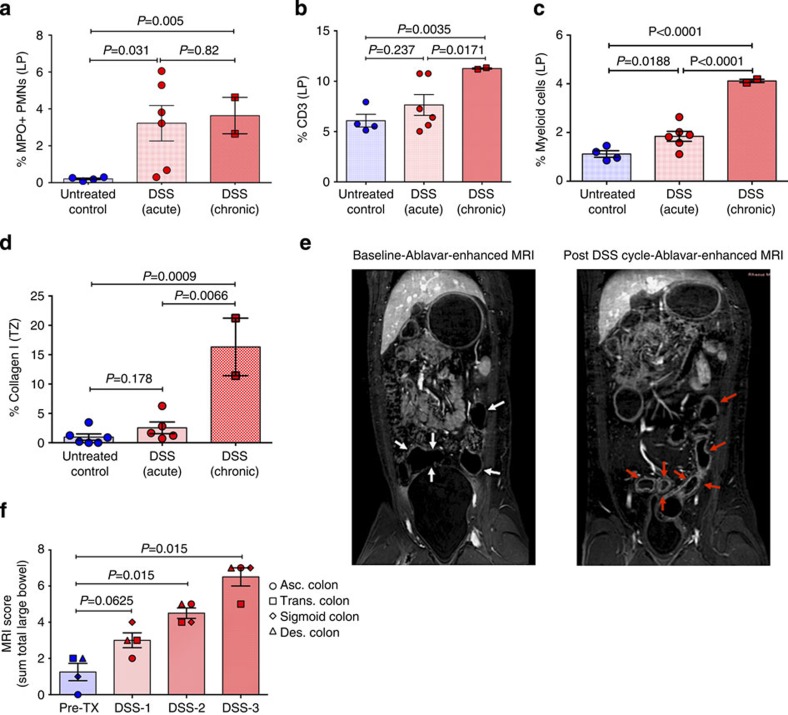
Features of DSS-induced chronic colitis and noninvasive MRI evaluation. Quantification of the percent area of the colon (all segments) that is occupied by (**a**) MPO+ PMNs, (**b**) CD3+ T cells and (**c**) CD68/CD163+ myeloid/macrophages in untreated control and DSS-treated acute and chronic colitis RMs. (**d**) The percent area of the AxLN that is occupied by collagen 1 in untreated control and DSS-treated acute and chronic colitis RMs. (**e**) Prominent inflammatory changes appear as enhancement with Ablavar (red arrows) in the colon after DSS treatment, as well as evident increased thickness of the colonic wall, stenosis and narrowing of colonic cavity. (**f**) Summation of the qualitative MRI scoring for each GI tract segment from both RMs showing the progression in the severity of colitis. *P* values are based on the Mann–Whitney test. Untreated control RMs (blue circles) *n*=4 (**a**–**c**) to 6 (**d**), acute DSS-treated RMs (red circles) *n*=6, and chronic DSS-treated RMs (red squares) *n*=2. Bar graphs show group means ±s.e.m. with individual animal data points shown.

Here we show that multiple, repeat cycles of DSS induce sustained colitis with gross lesions evident throughout the colon and significant PMN infiltration (Supplementary Fig. 8 and Fig. 5a). We also observe an increased density of CD3+ T cells and macrophages within the inflamed bowel indicating a chronic inflammatory state (Fig. 5b,c). Strikingly, induction of chronic colitis and resulting systemic inflammation is sufficient to induce lymphoid tissue fibrosis similar to what is observed as a hallmark histopathologic feature in pathogenic HIV/SIV infections (Fig. 5d). Importantly, correlative longitudinal MRI observations show evidence of significant and progressive inflammation within all compartments of the colon (Supplementary Table 2 and Fig. 5e,f). Collectively, our results demonstrate the establishment of both acute and chronic colitis models in NHP and show that MRI with a reversible albumin-bound blood pool contrast agent may be a useful, noninvasive method to localize and monitor colitis.

### DSS-induced disease marker expression in SIV+ AGMs

**Figure 6:**
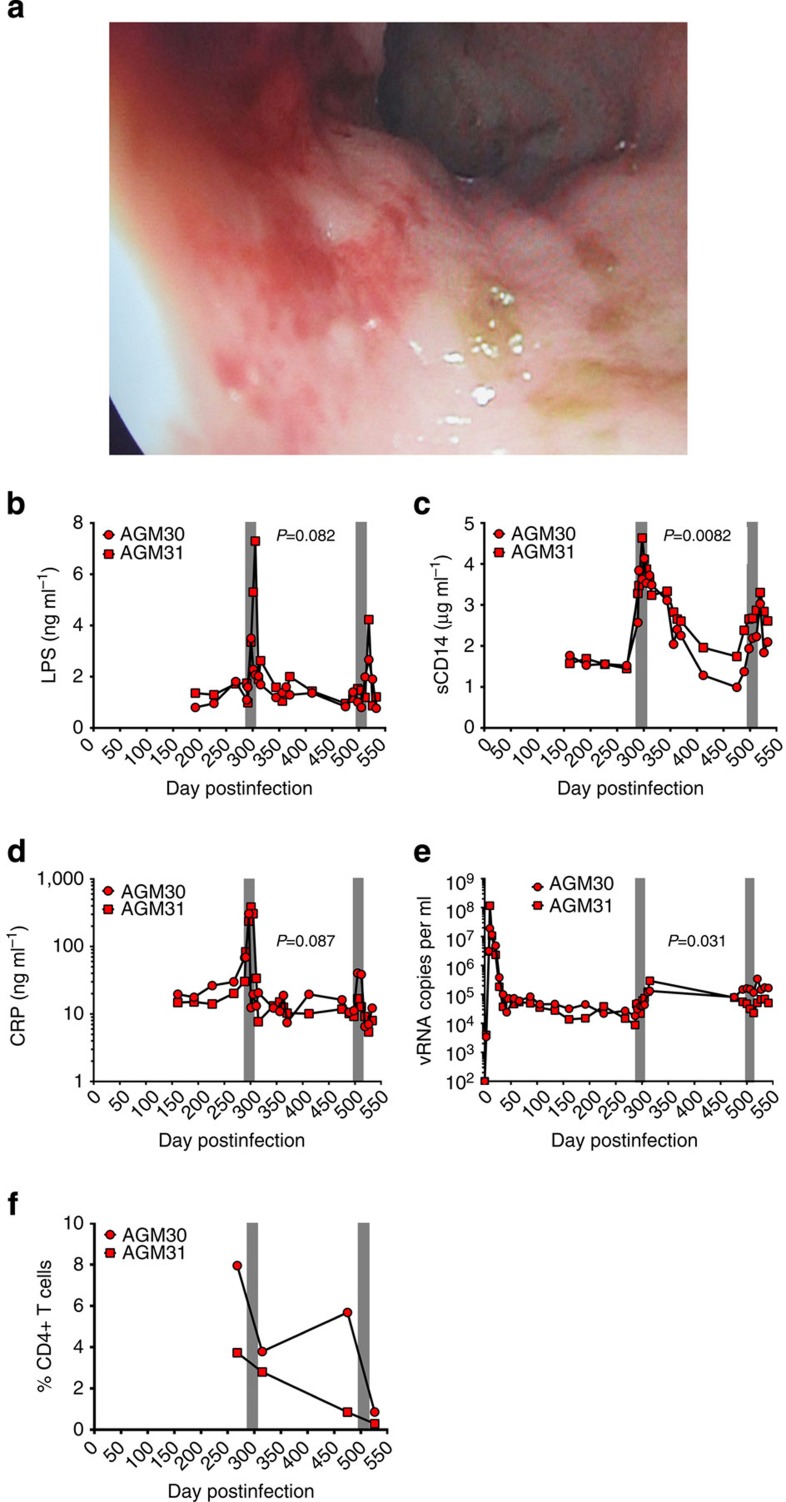
Impact of DSS treatment on chronically SIV+ AGMs. (**a**) Colonoscopy image showing multifocal mucosa thickening, redness and ulcerations. Longitudinal changes in plasma levels of LPS (**b**), sCD14 (**c**), CRP (**d**) and viral loads (**e**), and frequency of mucosal CD4+ T cells (**f**) in SIV+ AGMs (*n*=2) before and after two DSS treatments (shown by grey bars). *P* values were derived from repeated measures analysis of variance, linear hierarchical mixed effects models as described in methods section.

Natural hosts of SIV display limited-to-no chronic inflammation or immune activation and do not typically progress to AIDS despite high levels of viral replication during chronic infection. This has been interpreted as indicating that viral replication may be necessary, but is not sufficient for development of AIDS, with chronic immune activation representing a potential key driver of progressive pathogenesis. Notably, natural hosts of SIV maintain preserved GI tract integrity and mucosal immunity in the chronic phase of infection, without evidence of microbial translocation or associated inflammation and immune activation^11^,^39^,^40^,^41^,^42^,^43^,^44^. Therefore, to investigate the interconnection between microbial translocation, inflammation, immune activation and pathologic consequences in the setting of SIV infection of a natural host species, we experimentally induced damage to the intestinal epithelial barrier by administering two DSS treatments separated by about 200 days to two chronically SIV-infected AGMs. Here we show that DSS treatment of SIV+ AGMs results in colitis evidenced by gross examination during colonoscopy (Fig. 6a). In addition, each DSS treatment results in increased translocation of microbial products (LPS), immune activation (sCD14) and inflammatory markers (acute-phase protein; CRP) in the plasma of SIV+ AGMs (Fig. 6b–d). At the end of the first DSS treatment a significant increase in the plasma viral loads is observed and is accompanied by a partial depletion of rectal CD4+ T cells (Fig. 6e,f). The viral loads are then maintained at increased levels between the two treatments and mucosal CD4+ T cells continue to decline, reaching the maximum of depletion at the end of the second DSS treatment. These results show that DSS-induced GI tract damage in SIV+ AGMs leads to systemic effects that are reminiscent of progressive SIV disease in infected non-natural hosts.

## Discussion

Chronic inflammation and immune activation play a central role in driving disease progression in HIV infection. GI tract mucosal damage leading to microbial translocation is suggested to be an important underlying cause of this heightened state of activation of the immune system in this disease and in associated NHP models of AIDS^1^,^10^,^11^,^30^,^45^. However, direct evidence that GI tract damage leading to microbial translocation independent of lentiviral infection leads to systemic inflammation and immune activation, specifically T-cell activation, has been lacking. We developed an experimental NHP model of chemically induced colitis, in the absence of lentiviral infection, to test the hypothesis that GI epithelial damage leading to microbial translocation results in systemic inflammation and immune activation. Adapting an established small animal colitis model, we show for the first time an experimental NHP colitis model that recapitulates salient features of HIV/SIV infections, including GI tract damage and local and systemic microbial translocation, inflammation and immune activation.

Chemical induction of both acute and chronic colitis has widely been used in small animal models to study IBD pathogenesis^46^,^47^, and while these models have been an important resource and tool for the field, these small animal models do have important limitations and do not entirely represent the complexity of the human disease^21^. Thus, the development of a large animal model of colitis that more closely mirrors human physiology and immunity may be more clinically relevant and translatable to humans. In our study, we were able to induce both acute and chronic colitis in NHPs with a substantially lower concentration of DSS (4- to 20-fold less) than what is used in rodent studies. Similar to the studies of colitis induction in mice and findings in IBD in humans^18^, we also observed in DSS-treated macaques significant histologic damage to the large intestine that was associated with PMN infiltration, GI tract epithelial damage, and associated microbial translocation, inflammation and immune activation; all in the setting of clinically mild symptoms^48^,^49^.

Our previous studies of the timing and magnitude of GI tract damage and microbial translocation in SIV-infected RMs showed that GI tract damage and resulting microbial translocation occur early in infection with sustained increased levels of damage in the chronic stage leading to abundant systemic microbial products detected in peripheral lymph nodes^11^. While these and other studies only showed associations of GI tract damage and inflammation and immune activation, here we directly demonstrate, in the absence of lentiviral infection, that experimentally induced GI tract damage independently results in local and systemic microbial translocation, inflammation and immune activation in DSS-treated RMs. Similar to SIV-infected RMs, the bacterial species represented in microbial products found in the tissues in DSS-treated, uninfected animals show distinct differences in community structure compared with the GI tract, with an enrichment of Proteobacteria and Actinobacteria and a reduction of Firmicutes. These data warrant new studies of the effect of these bacterial phyla in systemic inflammation/immune activation and their role in IBD. Further studies of DSS-induced colitis and the associated systemic inflammation and immune activation, in the setting of experimentally induced immunodeficiency, still in the absence of lentiviral infection, may provide further insights into the mechanisms underlying AIDS virus pathogenesis.

DSS treatment of chronically SIV-infected AGMs, a natural host species for SIV in which infection does not induce excessive immune activation and inflammation and typically does not progress to AIDS, demonstrated that superimposed DSS-induced GI tract damage with resultant microbial translocation may be sufficient to drive systemic inflammation/immune activation, which augmented viral replication and mucosal CD4 T-cell depletion. It will be interesting to determine whether inducing prolonged colitis in chronically SIV+ AGMs will alter the balance of this disease in these natural hosts to a more ‘progressive’ phenotype typical of pathogenic disease.

Our NHP colitis model recapitulates many of the hallmark features of the disease state in chronically SIV+ RMs and demonstrates that GI tract damage leading to microbial translocation is sufficient to drive local and systemic inflammation and immune activation in the absence of SIV infection. We also show that gut damage also significantly increases the levels of immune activation and inflammation markers in chronically SIV-infected AGMs in which these parameters are usually maintained at baseline levels.

The implications of this study go beyond the demonstration of how GI tract damage in HIV/SIV infections may lead to systemic inflammation and immune activation. For the first time, we introduce a NHP model of acute and chronic colitis and show how a noninvasive MRI strategy we developed for evaluating the severity of colitis correlates with gold standard histopathologic findings and may have broad applicability in noninvasive monitoring IBD, HIV and colon cancer patients. Therefore, this novel NHP colitis model sets the stage for future studies on MRI clinical monitoring of IBD using gadolinium-based magnetic resonance contrast agents for intestinal microvasculature leakage, as well for evaluation of novel new therapeutic intervention strategies in a model that the features of which should be more translatable to human patients than small animal models of colitis.

## Methods

### Animals and ethics statement

All RMs (*Macaca mulatta*) in this study were mature and of Indian origin consisting of both male and female animals ranging in age from 2 to 7 years, and were housed at the National Institutes of Health (NIH) in accordance with the Association for the Assessment and Accreditation of Laboratory Animal Care standards and all procedures were performed according to the protocols approved by the Institutional Animal Care and Use Committee of the National Cancer Institute (Assurance #A4149-01) as previously reported^50^. In total, 12 RMs were used across the various groups in our DSS studies (6 RMs for SIV-uninfected acute colitis model, 2 RMs for SIV-uninfected chronic colitis model and MRI and 4 RMs for SIV-uninfected untreated controls). Animal numbers were chosen based on previous experience of necessary group sizes needed to see effects after treatment and MRI capacity. In addition, we utilized as control tissues from up to 20 RMs from previous retrospective studies where specimens of large bowel, peripheral LNs or both were collected (6 RMs for SIV-untreated controls and 14 RMs for chronic SIV+). Before use, all animals were tested and confirmed seronegative for Macacine herpes virus 1 (herpes B), SIV, simian T-lymphotropic virus and simian retrovirus, and were negative for simian retrovirus by PCR as well. In addition, before study, all animals were treated with enrofloxacin (10 mg kg^−1^ once daily for 10 days), paromomycin (25 mg kg^−1^ twice daily for 10 days) and fenbendazole (50 mg kg^−1^ once daily for 5 days) followed by weekly faecal culture and parasite exams for 3 weeks to ensure they were free of common enteric pathogens. A post-treatment period of at least 4 weeks was chosen to allow time for stabilization of the microbiome, although not formally tested in this study. After study completion, all 12 animals in our prospective study were euthanized and a detailed necropsy performed with histological evaluation of GI tract and peripheral lymphoid tissues.

Caribbean AGMs (*Chlorocebus sabaeus*, *n*=2) were housed and maintained at the University of Pittsburgh according to the standards of the Association for Assessment and Accreditation of Laboratory Animal Care, and experiments were approved by the University of Pittsburgh Institutional Animal Care and Use Committee (IACUC). These studies were covered by two IACUC protocols: 0907039/12080831 Animal Model for SIV Infection Control and 0911844/12121250 Pathogenesis of SIV in African green monkeys. The animals were fed and housed according to regulations set forth by the *Guide for the Care and Use of Laboratory Animals* and the Animal Welfare Act.

### Dextran sulfate sodium dosing and duration

Dextran sulfate sodium (0.25–1% (w/v) DSS, molecular weight 35,000–50,000 Da; MP Biomedicals, Solon, OH, Catalogue Number: 180558) was prepared in sterile distilled water with non-coloured Kool Aid to enhance intake, filtered and provided in drinking water bottles. DSS–water consumption was monitored and changed out three times a day, with a total DSS–water dosing of 100 ml kg^−1^, which was in accordance with the guidelines previously set for NHP daily fluid consumption^27^,^28^. If the DSS was completely consumed before water changes, then the animals were supplemented with normal water. If the DSS was not consumed before the bottle change times, the remaining unconsumed DSS–water was added to the fresh DSS–water bottle for that day; however, new water bottles were provided each morning regardless if the animals completely consumed their last dose from the night before. Most animals consumed the entire daily volume of DSS–water for the treatment duration, however, some animals failed to ingest the prescribed dose and some animals consumed each dose more rapidly than others, which may be reflected in some of the animal-to-animal variability seen in our measures of colitis.

DSS dose optimization was performed by treating two healthy SIV-negative RMs with 1.0% DSS in drinking water for 5 days, which was considered a conservative concentration and duration of DSS based on small animal studies^21^. DSS-treated and two untreated controls underwent colonoscopy with collection of colonic biopsies to evaluate both the gross and histological extent of colitis. Owing to the severity of colitis in animals treated with 1% DSS for 5 days, we then tested 0.25% DSS for 7 days and then for 10 days, which yielded optimal mild-to-moderate colitis with few overt clinical symptoms, and thus this dose (4- to 20-fold less DSS than in most murine DSS models published) and duration were chosen for subsequent experiments. Chronic colitis was induced by treating SIV-uninfected RMs with multiple cycles of DSS (1 cycle=14 days treated with 0.25% DSS followed by 14 days off DSS). Animals received MRI and colonoscopy after each cycle of DSS and after the last cycle of DSS were euthanized followed by a detailed necropsy and histological evaluation.

### Immunohistochemistry and quantitative image analysis

Tissue samples were collected at necropsy and immediately placed in cold media (for cell isolation and fluorescence-activated cell sorting analysis) or fixed in freshly prepared neutral buffered 4% paraformaldehyde for 24 h at room temperature. After fixation for 24 h, fixative was replaced with 80% ethanol and tissues were paraffin embedded. Immunohistochemistry (IHC) for *E. coli*, claudin-3, Mx1, MPO, CD3, Ki67 and collagen I was performed as previously described^11^,^51^,^52^,^53^.

IHC was performed using a biotin-free polymer approach (Golden Bridge International, Inc.) on 5-μm tissue sections mounted on glass slides, which were dewaxed and rehydrated with graded alcohols to double-distilled H_2_O. Heat-induced epitope retrieval was performed by heating sections in 0.01% citraconic anhydride containing 0.05% Tween-20 or 1 × Diva buffer (Biocare Medical) in a pressure cooker set at 122–125 °C for 30 s. Slides were incubated with blocking buffer (tris-buffered saline (TBS) with 0.05% Tween-20 and 0.25% casein) for 10 min. For IHC of myeloid cells, slides were loaded on an IntelliPATH autostainer (Biocare Medical) and stained with optimal conditions determined empirically consisting of a blocking step using blocking buffer for 10 min, an endogenous peroxidase block using 1.5% (v/v) H_2_O_2_ in TBS (pH 7.4) for 10 min, incubation with mouse anti-CD68 (1:400; clone KP1, Dako), mouse anti-CD163 (1:400; clone 10D6; Novocastra/Leica) and rabbit monoclonal anti-CD4 (1:200; clone EPR6855; Epitomics, Inc.) diluted in blocking buffer for 1 h at room temperature, washed with TBS containing 0.05% Tween-20 (TBS–Tw) and detection using a biotin-free polymer approach consisting of Mouse Polink-1 AP (Golden Bridge International, Inc.) for 30 min at room temperature followed by Rabbit Polink-1 HRP (Golden Bridge International, Inc.) for 30 min at room temperature. Sections were washed and first incubated with Impact DAB (3,3′-diaminobenzidine; Vector Laboratories) to develop the CD4, washed and developed with Warp Red (Biocare Medical, Inc.) to develop macrophage/myeloid cells and to mask the faint CD4 expressed on APCs to distinctly identify CD4+ T cells from myeloid cells.

For IHC of P-STAT1 and P-STAT3, slides were incubated in blocking buffer, then incubated overnight at 4 °C with P-STAT1 rabbit mAb (Tyr701; clone 58D6; Cell Signaling Technology; Catalogue No. 9167) and P-STAT3 rabbit mAb (Tyr705; clone D3A7; Cell Signaling Technology; Catalogue No. 9145) diluted 1:200 in blocking buffer. Tissue sections were washed, and the Rabbit Polink-2 staining system (Golden Bridge International, Inc) was utilized to develop signal according to the manufacturer’s recommendations. Sections were developed with Impact DAB.

All slides were washed in ddH_2_O, counterstained with haematoxylin, mounted in Permount (Fisher Scientific) and scanned at high magnification ( × 200) using a whole-slide scanning microscope (Aperio AT2 System, Aperio Technologies), yielding high-resolution data from the entire tissue section. Representative regions of interest (500 × 500 μm) were extracted from these whole-tissue scans for quantification.

The percent area of the tissue anatomical site of interest (that is, lamina propria of the GI tract, T-cell zone of the LN) that stained for the protein or cell type of interest was quantified using Photoshop CS5 or CS6 (Adobe Systems, San Jose, CA) and Fovea (Reindeer Graphics, Ashevile, NC) image analysis tools as previously described^11^,^52^,^53^. The percent GI damage was calculated by first measuring the linear length of the colon with damaged epithelial integrity using the pen tool in ScanScope (Aperio Technologies) on colon segments stained for claudin-3 and dividing this value by the measured linear length of the entire colon segments in each tissue section (% damage=(damaged GI tract length/total GI tract length) × 100). The linear length of the colon analysed for SIV-uninfected control RMs was on average 64.5 mm (s.e.m.=12.2), for SIV-uninfected DSS-treated RMs was 74.5 mm (s.e.m.=17.2) and for chronic SIV-infected RMs was 170.9 mm (s.e.m.=28.3).

### Histopathology scoring

On the basis of the histologic changes in PMN infiltration and damage to the intestinal epithelium on haematoxylin and eosin-stained tissue sections, the lesions were categorized into four grades that were modified from Geboes *et al.*^29^ by a pathologist blinded to the groups. Grade 0: normal colorectal mucosa. Grade 1: colorectal mucosal structure is well preserved, but neutrophils are infiltrated among crypts in the lamina propria from bottom to top of the mucosa, other cells such as fibroblasts and increased number of blood capillary vessels can also be found in the lamina propria. Goblet cells in some of the crypts were lost; the architecture of the GI tract was mostly preserved. Grade 2: normal colorectal mucosal structure is replaced by a large number of neutrophils, fibroblasts and newly formed blood capillary vessels. The columnar crypts are distorted or lost but the integrity of top layer of epithelial cells is present, albeit histologically abnormal. Grade 3: type 2 lesion plus disrupted top layer of epithelial cells, ulceration and necrosis. The inflammatory cells may extend into submucosa, smooth muscle and serosal layer.

### Flow cytometry

Peripheral blood mononuclear cells were isolated from whole EDTA blood by Ficoll-Paque Plus (GE Healthcare) gradient centrifugation. Mononuclear cell preparations of intestinal biopsy and necropsy tissues were obtained by mechanical disruption and enzymatic dissociation followed by lymphocyte separation by centrifugation through Ficoll-Paque Plus (GE Healthcare), as described previously^54^. Freshly isolated cells were immunophenotyped using the following antibody panel: CD4 Pacific Blue (clone OKT4; BioLegend; 1:50); CCR5 PE (clone 3A9; BD Biosciences; 1:20); CD28 ECD (clone CD28.2; Beckman Coulter, 1:20); CD95 PE-Cy5 (clone DX2; BD Biosciences; 1:10); CD8 PE-Cy7 (clone SK1: BD Biosciences; 1:100); CD38 APC (clone OK10; NIH Nonhuman Primate Reagent Resource; 1:25); CD3 APC-Cy7 (clone SP34-2; BD Biosciences; 1:100); and Ki67 FITC (clone B56; BD Biosciences; 1:20). Approximately 100,000 CD3+ T cells were acquired for each sample and data analysis was performed using FCS Express software. All dilutions are from the manufacturer’s stock and based on 100 μl total staining volume.

### Longitudinal evaluation and quantification of colitis by MRI

All MRI scans were performed in the Molecular Imaging Clinic (B3B) at the NIH campus on a 3T Philips Achieva MRI instrument. The MRI protocol was adapted from human and animal studies of the bowel during inflammation. A total of 4–5 imaging sessions were performed utilizing two RMs per session, examined between 15:00 and 21:00 hours. All animals were anesthetized with ketamine (10 mg kg^−1^) and dexmedetomidine (25 μg kg^−1^) followed by intubation and administration of isoflurane (1–3%), monitored throughout the session by a veterinarian and recovered without incident. Colon dilatation was performed with a Foley catheter to block the anus and up to 180 ml of air inserted into the colon. A baseline three-dimensional T1-weighted sequence with fat suppression was acquired with a cardiac coil (Philips Medical Systems, Best). Then, the animals received 0.12 ml kg^−1^ (250 mM; containing 0.03 mM gadolinium) of gadofosveset trisodium (Ablavar, Lantheus Medical Imaging) administered intravenously in conjunction with the Food and Drug Administration guidelines. Following intravenous injection of gadofosveset trisodium three phases of post-injection T1-weighted MRI were acquired. Magnetic resonance acquisition parameters are presented in Supplementary Table 2. Quantitative MRI scoring system for differences in the severity of colitis was determined by using the following MRI criteria for bowel inflammation modified from Friedrich *et al.*^55^ presented in Supplementary Table 3. If none of the criteria for inflammation was present, the segment was deemed not inflamed (grade 0). In the presence of one criterion, the segment was found to be mildly inflamed (grade 1). If two criteria were fulfilled, we diagnosed the segment as moderately inflamed (grade 2). When three or more of the MRI criteria for inflammation were present, the segment was judged severely inflamed (grade 3). Segmental evaluation: ascending colon, transverse colon, descending colon and sigmoid colon.

### Isolation of nucleic acids and 454 pyrosequencing

DNA was isolated and 454 pyrosequencing was performed as previously described^32^ Briefly, DNA was isolated from ∼3 mg of snap frozen tissue using the QIAmp DNA Stool Kit (Qiagen, Valencia, CA) following the manufacturer’s instructions. RNA was isolated from stool using Trizol (Life Technologies, Carlsbad, CA) following the manufacturer’s protocol with the addition of two to three additional phenol/chloroform extractions following the initial phase separation. DNA was isolated from tissues using the DNeasy Blood and Tissue Kit (Qiagen) following the manufacturer’s instructions. DNA and RNA were extracted from luminal swabs by tandem use of the RNeasy Mini and DNeasy Blood and Tissue Kits (Qiagen). Nucleic acids were washed off of the luminal swabs using buffer RLT with β-mercaptoethanol from the RNeasy kit. This volume was then used to extract RNA following the manufacturer’s protocol. Flow-through from the binding step and the RW1 buffer was saved and combined for use in DNA extraction. Collected flow-through was combined and precipitated with ethanol. The resulting pellet was treated with buffer ATL plus proteinase K from the DNeasy kit and used for DNA extraction.

For sequencing of 16S rDNA, amplicon libraries were prepared from sample DNA using Accuprime high fidelity Taq polymerase (Invitrogen, Carlsbad, CA) and universal primers flanking variable regions V1 (primer 27F; 5′-AGAGTTTGATCCTGGCTCAG-3′) and V3 (primer 534R; 5′-ATTACCGCGGCTGCTGG-3′). For each sample, the universal primers were tagged with unique sequences (barcodes) to allow for multiplexing/demultiplexing. PCR products were then purified using the Agencourt Ampure XP Kit (Beckman Coulter Genomics, Danvers, MA) and quantitated using the QuantIT dsDNA High-Sensitivity Assay Kit (Invitrogen). Approximately equivalent amounts of each PCR product were then pooled and purified with a Qiagen minElute column (Qiagen) into 30 μl TE buffer before sequencing on a 454 FLX instrument (Life Sciences, Branford, MA) using Titanium chemistry. Raw sequences of the V1–V3 hypervariable regions of the 16S rRNA gene were processed and analysed using Mothur v.1.31.2 (ref. 56). Sequences were trimmed of primer and barcode sequences (primer differences allowed, 2 bp, barcodes, 1 bp) to a minimum length of 200 bp and denoised using the Mothur implementation of PyroNoise^57^. Sequences were aligned using the SILVA database, and chimeras removed using UCHIME^58^. The taxonomic assignment was determined using the ribosomal database project (RDP) Classifier included in Mothur. Operational taxanomic units (OTUs) were defined at an identity cutoff of 97%. Metastats^59^ was used to detect differentially abundant OTUs controlling the false discovery rate (<0.05). To visualize clustering of communities among samples, distance matrices were generated using the Yue and Clayton theta (relative abundance) measures of dissimilarity and used in principal coordinates analysis plots.

### Plasma viral load quantification

SIVagmSab viral load quantification was performed with primers and probe: SIV-pol-standard-R (5′-GCTTCAACAGGAACTTAGCTGTTTC-3′), and SIV-pol-standard-Probe (5′-/56-FAM/CCAGCAGTG/ZEN/GTGGCAAAGGAGA/3IABkF/-3′). A second set of primers and probe mapping the *gag* sequences were synthesized, as follows: SIV-gag-standard-F (5′-ATAGCAGGGACCACTAGCACAAT-3′), SIV-gag-standard-R (5′-TCTTTGAATGGTTCCTTGGGTCC-3′), and fluorogenic SIV-gag-standard-Probe (5′-/56-FAM/ATAGCAGGG/ZEN/ACCACTAGCACAATAC/3IABkF/-3′). All primers and probes were synthesized by IDTDNA (Coralville, IA) and were used in a two-step real-time PCR assay, as described^60^,^61^,^62^. First, reverse transcription of RNA to single-stranded cDNA was performed using the SuperScript III First-Strand Synthesis SuperMix for qRT–PCR kit according to the manufacturer’s recommendations (Invitrogen). Real-time PCR was performed in MicroAmp Optical 96-well plates (Applied Biosystems, Branchburg, NJ) by mixing TaqMan PCR Master Mix (Applied Biosystems) with 5 μl isolated RNA in a 50-μl final reaction volume. Real-time PCR conditions were as follows: 15 min at 95 °C, followed by 45 cycles of 95 °C for 15 s and 60 °C for 1 min. Dilutions of all components were made using sterile RNase-free water. Data were collected and analysed using the PE Applied Biosystems software. RNA copies per well were adjusted to copies per millilitre of plasma. Samples were tested in duplicate, and the numbers of RNA copies were determined by comparison with a standard curve obtained using known amounts of SIV-*pol* or SIV-*gag* RNA standards. The detection limit of the SIVagmSab quantification assay was 100 copies per ml.

### C-reactive protein testing

CRP was measured using a monkey CRP ELISA kit (Life Diagnostics, PA) as per the manufacturer recommendations.

### Assessment of the levels of microbial translocation in AGMs

Plasma levels of sCD14 and LPS in AGMs were measured as previously described^9^,^10^. Several factors present in plasma have been shown to interfere with LPS measurements (LPS-binding protein, EndoCAb, HDL, plasma turbidity, proteins and triglycerides). Therefore, to minimize any possible interference, plasma samples were diluted fivefold with endotoxin-free water and then heated to 85 °C for 15 min to inactivate plasma proteins. Plasma LPS was quantified with a commercially available *Limulus* amebocyte lysate assay (Cambrex), according to the manufacturer’s protocol. Each sample was run in duplicate. sCD14 levels were measured using a quantitative sandwich enzyme immunoassay technique (Quantikine Human sCD14 Immunoassay, R&D Systems, Minneapolis, MN). The detection limit of this kit is 200 ng ml^−1^ and can range up to 5,000 ng ml^−1^ at a dilution factor of 1:200, with an interassay coefficient of variability of 7.19–10.9%.

### Luminex assay

Lab 1: cytokines in plasma were determined by a NHP-specific Luminex (Austin, TX) assay that detected 36 cytokines as described elsewhere^63^. These cytokines included: G-CSF, GM-CSF, GRO-α, IFN-α, IFN-γ, IL-1β, IL-1Ra, IL-2, IL-4, IL-5, IL-6, IL-7, IL-8, IL-9, IL-10, IL-12 (p40), IL-12 (p70), IL-13, IL-15, IL-17, IL-18, IL-23, IP-10, MCP-1, MDC, MIP-1α, MIP-1β, perforin, RANTES, sCD40L, sFASL, TGF-α, TGF-β, TNF-α, TNF-β and VEGF. Lab 2: Luminex was performed on undiluted plasma samples using the Milliplex MAP Non-Human Primate Cytokine Magnetic Bead Multiplex Assay (Darmstadt, Germany) using the following bead-based antibodies: G-CSF, GM-CSF, IFN-**γ**, IL-1ra, IL-1, IL-2, IL-4, IL-5, IL-6, IL-8, IL-10, IL-12/23 (p40), IL-13, IL-15, IL-17, IL-18, MCP-1, MIP-1α, MIP-1β, sCD40L, TGF-α, TNF-α, VEGF. Luminex analysis was performed on a Bio-Plex 200 system using Bio-Plex Manager Software (version 6.1.1; Bio-Rad).

### Statistical analysis

Data comparing the untreated control group to the DSS-treated group utilized the Mann–Whitney *U*-test, while analysis of pre- and post-DSS-treated sample comparisons utilized the paired *t*-test or Wilcoxon matched-pairs signed rank test. Study animals for the prospective study were selected for inclusion in the study based on pre-specified criteria, randomly assigned to groups and these selected animals contributed data to all analyses. No power calculations were performed for this study. Animals selected from our retrospective tissue bank were selected based on SIV infection status and availability of tissue specimens. Not all the retrospective animals were included in every analysis due to limited or inadequate tissue samples for the particular analysis performed. No randomization was used to assign groups. All quantitative image analysis was performed using a semi-automated, blinded approach. AGM data in this study were analysed with repeated measures analysis of variance, linear hierarchical mixed effects models, *t*-tests, non-parametric tests (for example, Wilcoxon rank sum, matched pairs signed rank) and standard graphical techniques^54^. Repeated measures analyses and mixed effects models take into consideration the correlation/covariation of responses within the same animal over time. CRP, LPS, pVL, sCD14 and mucosal CD4+ T cell data for two macaques were recorded for varying numbers of occasions, within four to six treatment phases. Appropriate variance ratios were calculated to test for questions of interest, in particular, for differences among phases across time^54^. In some instances, *post hoc* comparisons were made among particular subsets of phases. Probability values <0.10 but >0.05 (0.10<*P*<0.05) were considered marginally significant, and values <0.05 (*P*<0.05) were considered significant.

## Additional information

**How to cite this article:** Hao, X. P. *et al.* Experimental colitis in SIV-uninfected rhesus macaques recapitulates important features of pathogenic SIV infection. *Nat. Commun.* 6:8020 doi: 10.1038/ncomms9020 (2015).

## Supplementary Material

Supplementary Information Supplementary Figures 1-8 and Supplementary Tables 1-3
