# Supplementary material for: Experimental colitis in SIV-uninfected rhesus macaques recapitulates important features of pathogenic SIV infection
Source: Nat Commun. 2015 Aug 18;6:8020. doi: 10.1038/ncomms9020 (PMC4544774; doi:10.1038/ncomms9020)
Supplement: Supplementary Information — Supplementary Figures 1-8 and Supplementary Tables 1-3 [file ncomms9020-s1.pdf]

Supplementary Figures

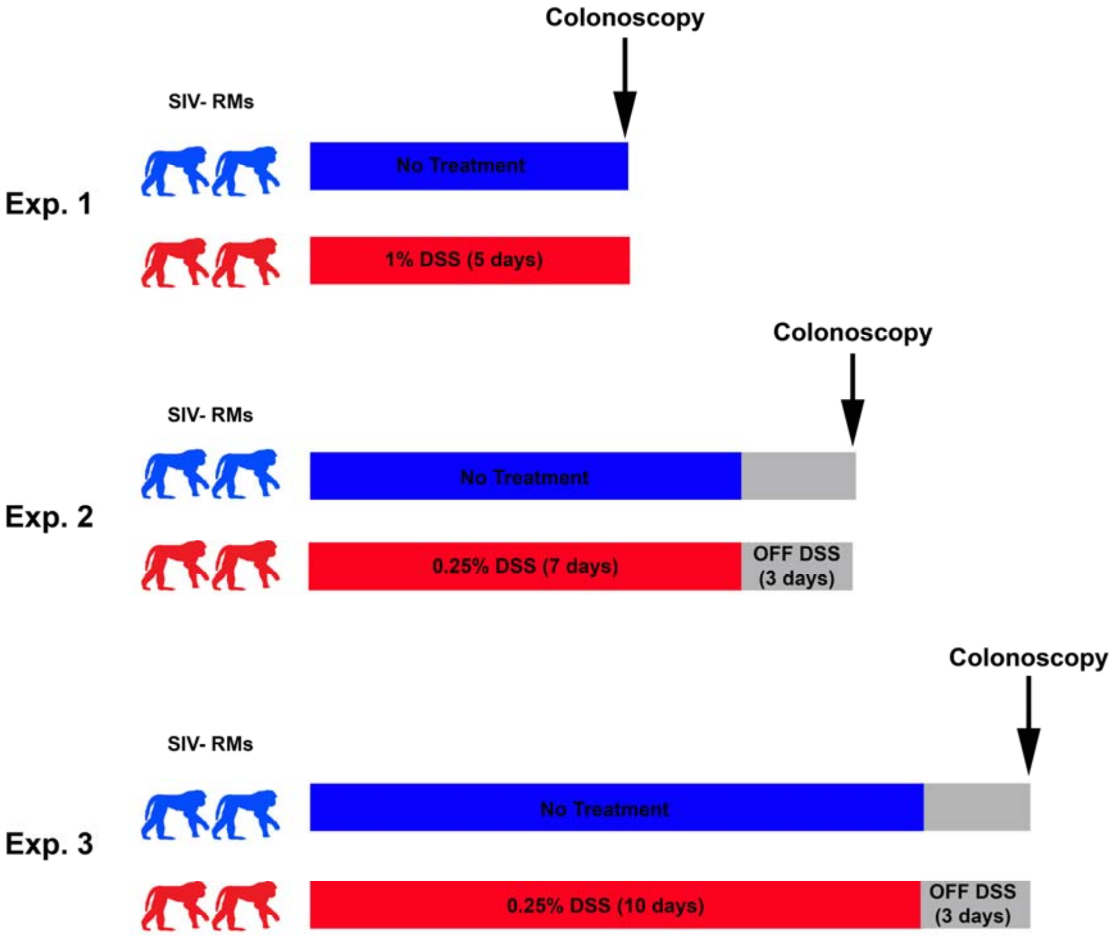

Supplementary Figure 1. DSS dose optimization strategy for acute colitis. Three rounds of experimental trials were performed to optimize the dose and duration of DSS treatment to achieve significant histologically evident colitis with mild clinical signs of disease. In experiment 2 and 3, colonoscopies were performed 3 days after the cessation of DSS.

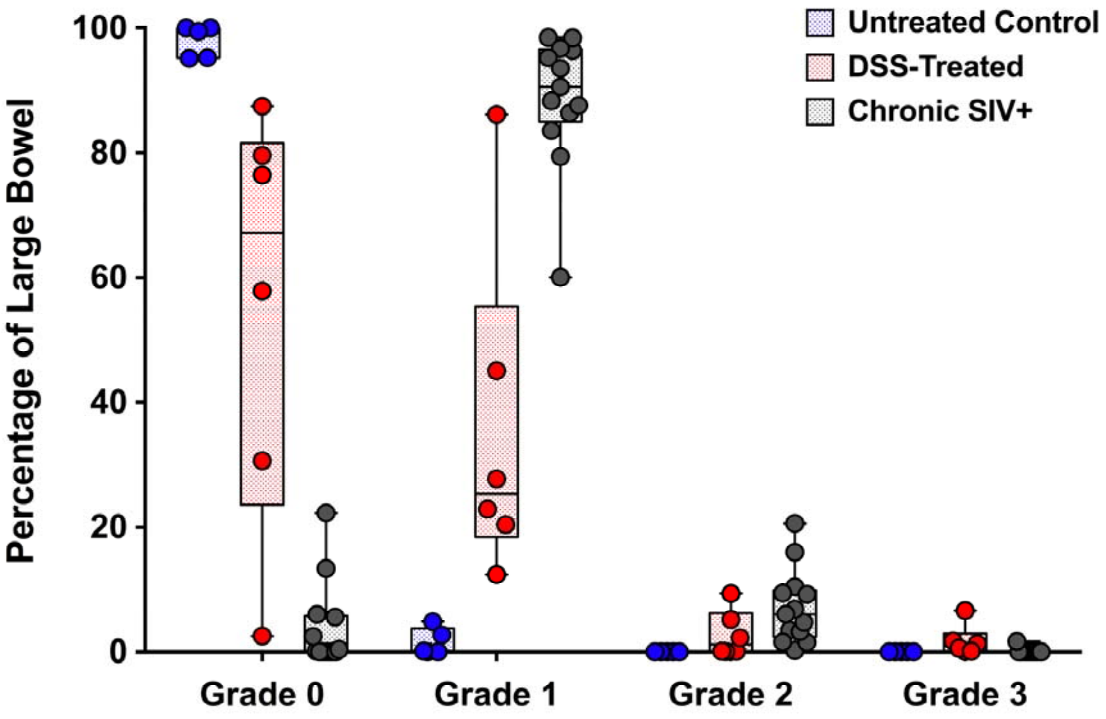

Supplementary Figure 2. Fraction of large bowel with each pathology score. After classifying tissues from all large bowel segments (ascending, transverse and descending colon and rectum) in to distinct pathology grades, we determined the proportion of the colon that consisted of each pathology grade in untreated control, DSS-treated, and chronic SIV+ RMs. Untreated control RMs (blue circles), acute DSS-treated RMs (red circles), and chronic SIV+ RMs (grey circles).

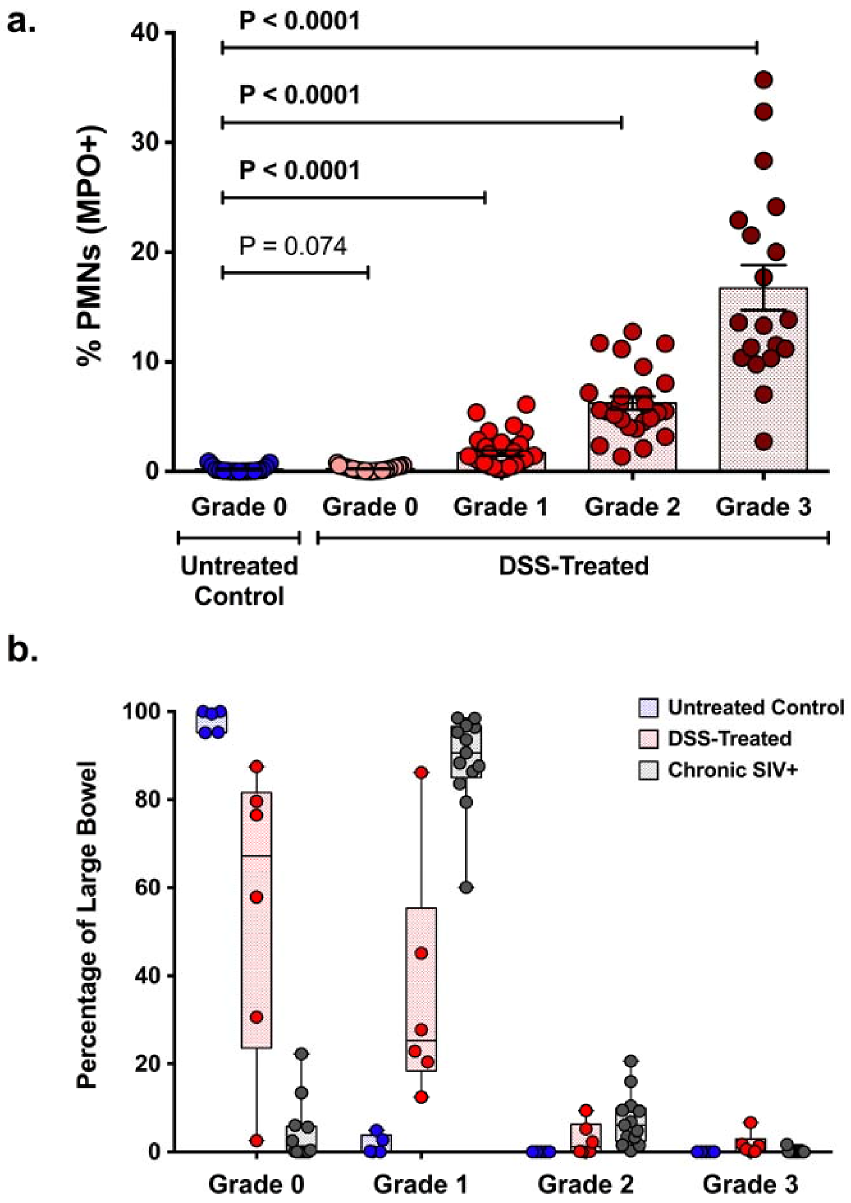

Supplementary Figure 3. Quantification of neutrophil (PMN) infiltration within each colon segment and pathology grade. (*a*) Colon tissues were stained for MPO and the percent area of PMNs within each distinct pathology grade was quantified. Each data point represents a distinct area of the colon with that particular pathology grade quantified. Notice the progressive increased infiltration of PMNs from grade 1, 2 to 3 colitis, but no difference in grade 0 between untreated control and DSS treated. (*b*) Quantification of the percent area of each colon segment that is occupied by MPO+ PMNs. P values are based on the Mann-Whitney test. Untreated control RMs (blue circles) n=5, acute DSS-treated RMs (red circles) n=6 and shades of red, and chronic SIV+ RMs (grey circles) n=13. Box plot graphs show group means with maximum and minimum values with individual animal data points shown.

a.

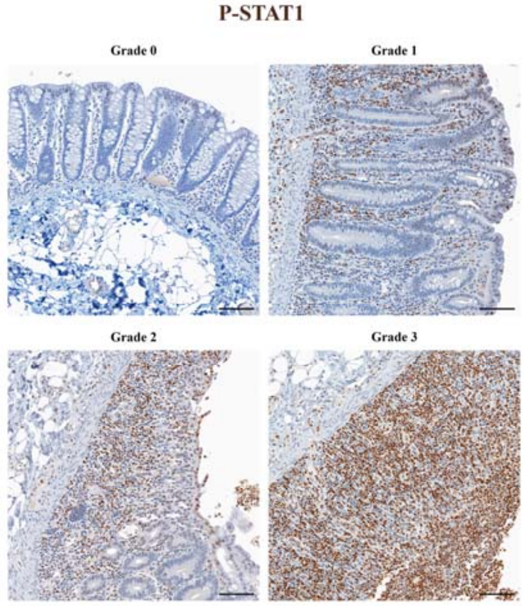

b.

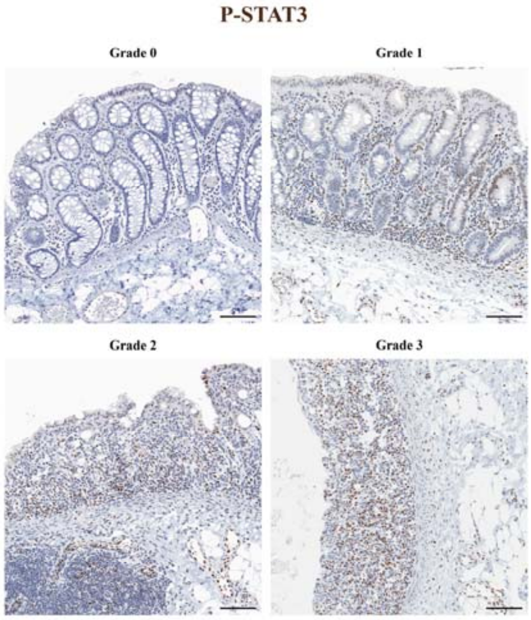

Supplementary Figure 4. Increased local inflammation (P-STAT1 and P-STAT3 expression) in colon following DSS treatment. Representative images of (*a*) P-STAT1 and (*b*) P-STAT3 stained colon showing limited inflammation in normal grade 0 (untreated control RM) colon and increased inflammation in grade 1-3 (DSS-treated RM) lesions. Notice the increased P-STAT1 and P-STAT3 expression levels in grade 1, 2 and 3 lesions associated with increased severity of inflammation. Scale bar = 100 μm.

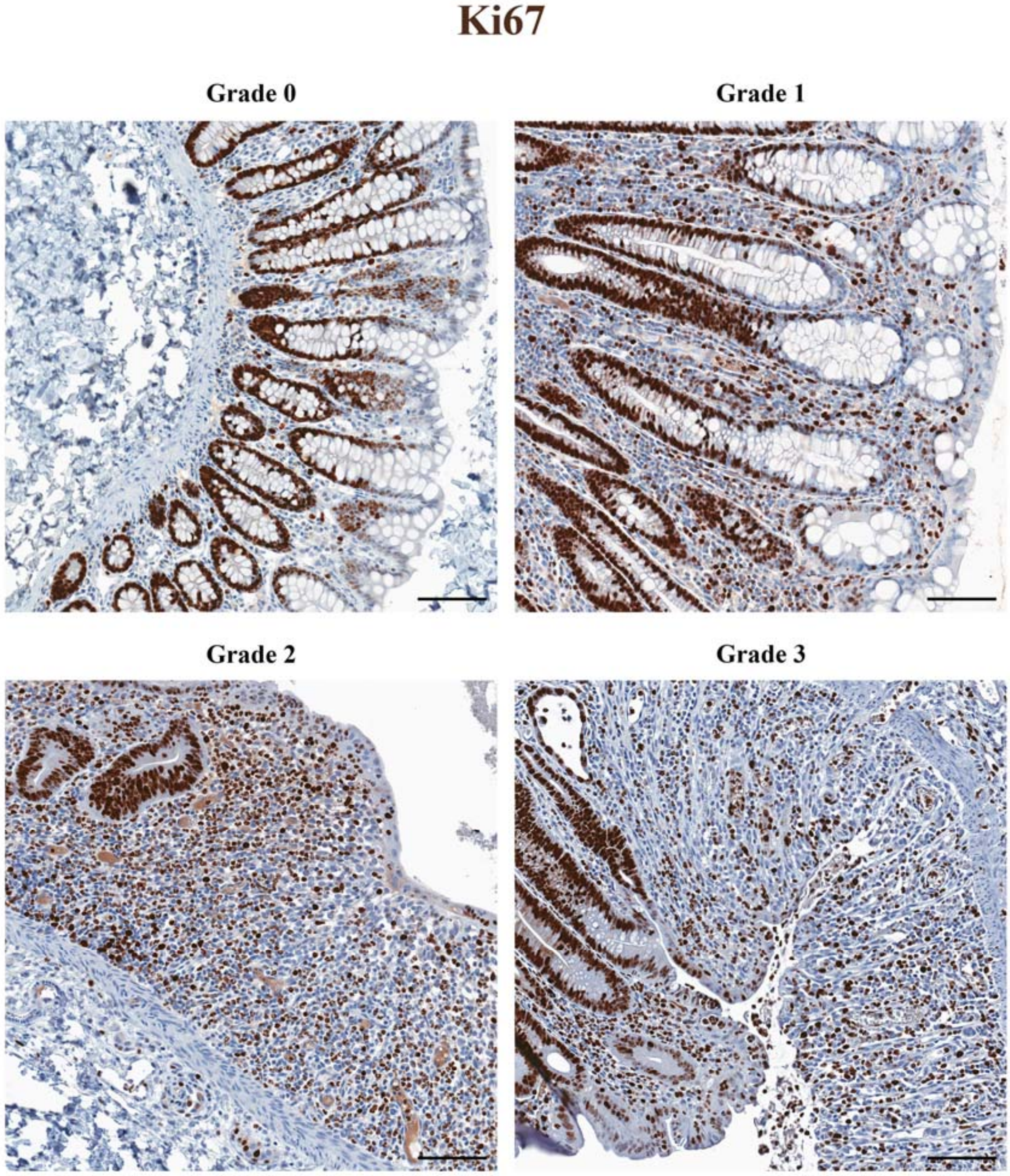

Supplementary Figure 5. Increased local immune activation (Ki67 expression) in colon following DSS treatment. Representative images of Ki67 stained colon showing limited immune activation in normal grade 0 (untreated control RM) colon and increased immune activation in grade 1-3 (DSS-treated RM) lesions. Notice the increased number of Ki67+ cells in the LP of DSS-treated RMs in grades 1-3 compared with grade 0 from untreated control animals. Scale bar = 100 μm.

a.

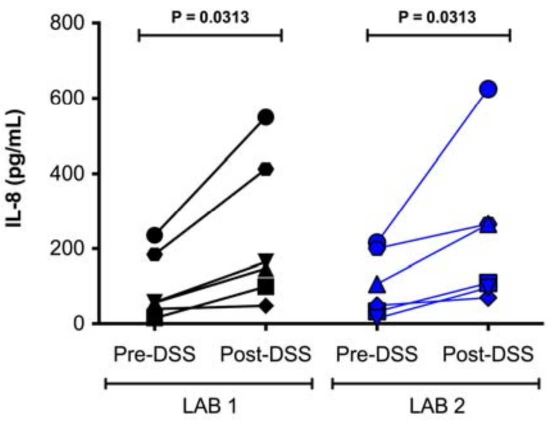

b.

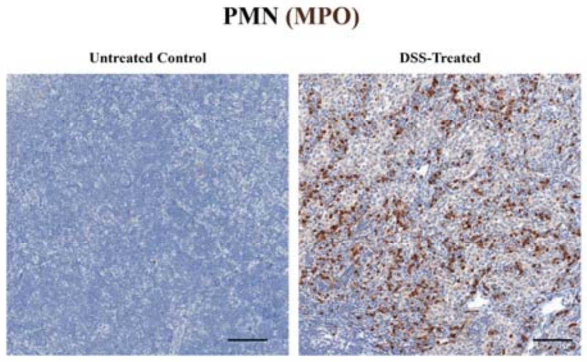

c.

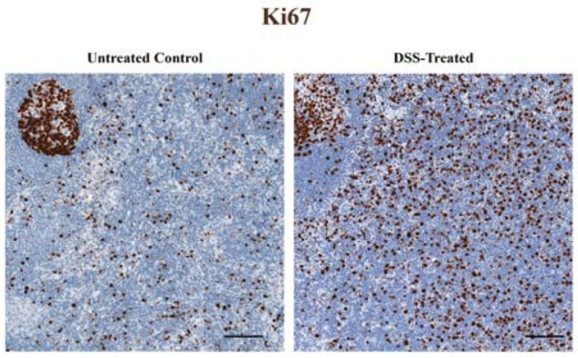

Supplementary Figure 6. Increased systemic inflammation and immune activation following DSS treatment. (*a*) Longitudinal quantification of IL-8 in the plasma pre- and post-DSS treatment (n=6) by Luminex from two independent laboratories. P values are based on the Wilcoxon matched pairs test. Representative images of (*b*) MPO+ and (*c*) Ki67+ stained AxLNs showing limited inflammation and immune activation in untreated control RMs and increased inflammation and immune activation in DSS-treated RMs. Scale bar = 100 μm. Symbols represent individual animals and distinct colors represent independent measures from two laboratories.

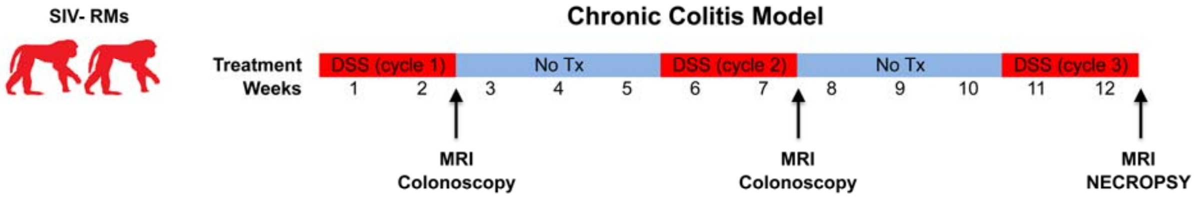

Supplementary Figure 7. Chronic colitis DSS dosing strategy. Schematic outlining the strategy for inducing chronic colitis by treating with multiple cycles of DSS (1 cycle was 14 days on DSS (0.25%) followed by 14 days off DSS).

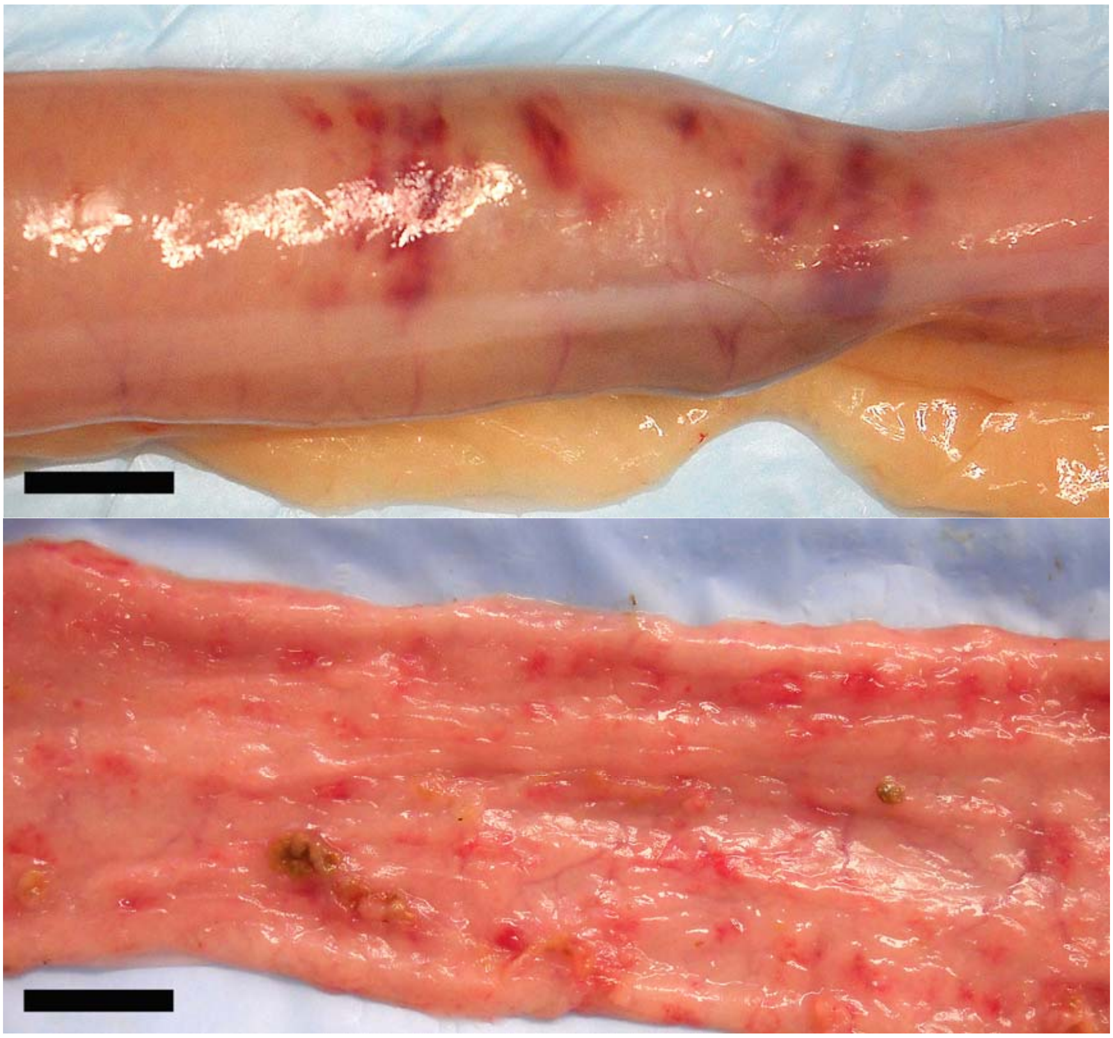

Supplementary Figure 8. Chronic colitis after three cycles of DSS treatment. Photomicrographs showing the gross lesions in the transverse colon before (top image) and after (bottom image) opening along the teniae coli ligament. Notice the multi-focal lesions with thickened mucosa, redness, and ulcerations. Scale bar = 1 cm.

Supplementary Tables

Supplementary Table 1. Pathological scoring system for quantifying the severity of colitis

Pathological Grade	Description
0	Normal mucosa with no evidence of inflammation.
1	PMN infiltration in LP; Intact epithelial barrier; GI structure preserved.
2	PMN infiltration in LP; Crypt distortion/loss; Goblet cell loss; Intact, but abnormal epithelial barrier.
3	PMN infiltration in LP and submucosa; Epithelial barrier loss; Ulceration/necrosis.

Supplementary Table 2. MRI acquisition parameters

Animal Weight	TR/TE (msec)	Matrix	Field of View (mm)	Flip Angle	Voxel Size (mm3)	Acquisition Time (sec)
~13 kg	6.1/2.9	508x200	306x200x180	25	0.6	400
~7 kg	6.7/3.1	600x160	300x140x110	25	0.35	365

Supplementary Table 3. MRI scoring system for quantifying the severity of colitis

Criteria
wall thickening > 3mm
bowel stenosis
increased contrast media uptake
enlarged local lymph nodes
local stranding of surrounding fat tissue

Scoring	Grade
0/5	No inflammation
1/5	Mild inflammation
2/5	Moderate inflammation
≥3/5	Severe inflammation
